# House and Garden

**Published:** 1882-11

**Authors:** 


### HOUSE AND GARDEN.

[From The Churchman.]

EVERGREENS are quite different in their nature and habits from deciduous trees, or those which shed their leaves. The latter are transplanted in March, April or November. The former in May, June, or from about the middle of August to the middle of September. Large experience, it may be said, *proves* the latter to be the best time. Growth is done for the season, and there are from two to three months before frost in which to establish new roots for the next year, favored by the long, cool nights. At the earlier season the tree is busy above ground, the long sunny days and dry winds aid in the exhaustion of the torn roots, which have enough to do to repair themselves, and the disappointing result is too often a flame-colored, dead tree.

Pines, we mean our beautiful, softly, lullaby-singing American white pine, is one of the most desirable of evergreens, but one of the most difficult to transplant. As may be seen by studying it in its home, the pine grows in a thin, dry soil, and therefore must not be pampered with richness. It flourishes in the gravelly roadside, and on dry hills, where the peeling off of a thin layer of mold brings to light a yellow earth; therefore prepare a soil poor enough for it. Take small specimens three feet high, or better, less, and shelter them well in March, whose cold, dry, searching, west winds are the hardest test of the endurance of evergreens.

Trees are as pleasant keepsakes of summer resorts as are photographs or Indian work. We have flourishing sugar-maples, six to twelve feet high, with pines, hemlocks, larches and native spruces, all brought in hand, by night express trains, at different times, from Lake Memphremagog, when they were but from six inches to a foot high.

Large trees, successfully transplanted, gain a highly gratifying amount of time in shading a bare, new place. A pair of handsome elms from an abandoned nursery, costing five and ten dollars, lived, and were a satisfying investment; and so were maples, six inches in diameter, for a wonder, successfully moved in June, when almost fully leaved out, being trees from the roadside, where a large ball of compact earth could be taken with them. Such trees are well prepared for moving in autumn, by loosening them, and then by moving them in winter with a large ball of frozen earth. They should be set deep and with the richest earth at the bottom of the hole to feed the roots.

Plants can be transplanted at any time, and even in full blossom, by the help of abundant water. This lesson was learned from a veteran Scotch gardener, whose literal circumspection was such that he seldom spoke without scanning at least two-thirds of the horizon as if for expected trespassers. Dig a hole, pour into it a whole pail of water; set your pansy, fuchsia, geranium, petunia or what not into it; heap up the earth around the plant, and it will go on without drooping, as if nothing had happened.

